# Enhancing Selectivity of Protein Biopharmaceuticals in Ion Exchange Chromatography through Addition of Organic Modifiers

**DOI:** 10.3390/ijms242316623

**Published:** 2023-11-22

**Authors:** Bastiaan Laurens Duivelshof, Thomas Bouvarel, Sebastian Pirner, Vincent Larraillet, Alexander Knaupp, Hans Koll, Valentina D’Atri, Davy Guillarme

**Affiliations:** 1 School of Pharmaceutical Sciences, University of Geneva, CMU—Rue Michel Servet 1, 1211 Geneva, Switzerland; 2 Institute of Pharmaceutical Sciences of Western Switzerland, University of Geneva, CMU—Rue Michel Servet 1, 1211 Geneva, Switzerland; 3 Roche Diagnostics GmbH, Nonnenwald 2, 82377 Penzberg, Germany

**Keywords:** bispecific antibody, Fab fragment, ion exchange chromatography, monoclonal antibody, organic modifier, salt gradient, salt-mediated pH gradient

## Abstract

Charge heterogeneity among therapeutic monoclonal antibodies (mAbs) is considered an important critical quality attribute and requires careful characterization to ensure safe and efficacious drug products. The charge heterogeneity among mAbs is the result of chemical and enzymatic post-translational modifications and leads to the formation of acidic and basic variants that can be characterized using cation exchange chromatography (CEX). Recently, the use of mass spectrometry-compatible salt-mediated pH gradients has gained increased attention to elute the proteins from the charged stationary phase material. However, with the increasing antibody product complexity, more and more selectivity is required. Therefore, in this study, we set out to improve the selectivity by using a solvent-enriched mobile phase composition for the analysis of a variety of mAbs and bispecific antibody products. It was found that the addition of the solvents to the mobile phase appeared to modify the hydrate shell surrounding the protein and alter the retention behavior of the studied proteins. Therefore, this work demonstrates that the use of solvent-enriched mobile phase composition could be an attractive additional method parameter during method development in CEX.

## 1. Introduction

Therapeutic monoclonal antibodies (mAbs) have become a staple in the current pharmaceutical industry by providing clinical solutions for many highly abundant diseases and as alternative therapies to conventional small molecular drugs [1,2,3,4]. The large-scale production of mAbs is performed by using recombinant DNA techniques to produce high titers of the antibody in stable cell lines [5]. Inherent to this type of production is the heterogeneity that is found among the produced antibody products due to a combination of enzymatic and chemical post-translational modifications (PTMs) [6,7,8,9,10]. Product charge heterogeneity, resulting from these PTMs, is an important critical quality attribute (CQA) that requires careful characterization throughout the lifecycle of the antibody product to ensure safe and efficacious drug products [11,12,13,14,15].

Ion exchange chromatography (IEX) is considered a gold standard technique for the characterization of protein charge variants [16,17,18]. Due to the isoelectric point (p*I*) of most approved therapeutic mAbs being over 7, cation exchange chromatography (CEX) is the most popular chromatographic technique to separate acidic and basic variants from the main mAb product [19,20,21,22]. The acidic species have lower apparent p*I*s and are produced via modifications such as the deamidation of asparagine, lysine glycations, and the sialic acid content, while basic species have higher apparent p*I*s and are formed via C-terminal lysine presence, methionine oxidations, or incomplete cyclization of N-terminal glutamate [13,21]. In order to separate and elute these variants from the CEX column, classical non-denaturing salt gradients and pH gradients are used with combinations of buffers (e.g., MES, HEPES) and salts (e.g., NaCl). These gradients result in the disruption of the ionic interactions between the charged functional groups of the stationary phase material and the net surface charge of the protein species by providing either counter ions in the form of salts or by changing the net surface charge of the protein [23]. However, the classical CEX conditions are not compatible with mass spectrometry (MS) due to the presence of the non-volatile salts. 

An interesting alternative is the use of pH gradients that are created with volatile ammonium acetate and acetic acid and provide the possibility to directly hyphenate CEX separations with MS detection [24,25,26,27,28,29,30,31]. However, directly hyphenating IEX with MS often leads to a compromised chromatographic separation due to the limited buffering capacity when only volatile salts are being used in the mobile phase [28,32,33]. This poses an increasing problem in the rapidly maturing mAb market, as numerous new complex formats are being introduced, containing a broad range of physico-chemical characteristics making their separation more challenging [34,35,36,37,38]. These formats include dual targeting Fabs, multi-specific antibodies, and antibody–cytokine fusions with lower and higher p*I* variants but also a wide variety in sizes due to the presence of a fused partner or added domains [15,39]. Consequently, there is a growing need for improved selectivity in IEX methods for mAbs and other formats. 

Recently, the use of a salt-mediated pH gradient was introduced to improve the separation of mAbs with a wider range of p*I*s and was achieved by simultaneously increasing the ionic strength in the buffer composition and the pH of the solution [30,40,41,42]. This avoids a commonly encountered problem in IEX with linear pH gradients, where the retention of acidic mAbs is hampered by the higher ionic strength at low pH, and basic mAbs lack separation due to the low ionic strength at high pH that limits the band compression effect due to the absence of counter ions. This was solved by adding an additional salt gradient to modulate the ionic strength during the linear pH response and improved the resolution for mAbs with p*I*s of <7.3 and >9.0. In this study, we explore the possibility of extending the selectivity of a salt-mediated pH gradient by adding different types of additives. It is common practice to add organic modifiers when analyzing oligonucleotides (ONs) and other small organic ions in IEX as an additional method parameter to improve the selectivity. However, such an approach has never been applied to proteins until now. In the present work, the effect of several additives on the chromatographic performance in CEX will be evaluated. In addition, their use as a means to reduce the required ionic strength for the elution of the proteins will be explored. The new buffer compositions will be tested by analyzing a conventional mAb product with a complex charge profile as well as three bispecific antibodies (bsAbs) with broad product characteristics in terms of size and p*I*.

## 2. Results and Discussion

### 2.1. Evaluation of Buffer Performance

Before the introduction of solvents and/or additives to the mobile phase composition, benchmark experiments were performed using two commonly used buffer systems, namely a classical salt gradient buffer based on MES + NaCl and a salt-mediated pH gradient based on ammonium acetate (full descriptions of the recipes can be found in Section 3.3). The different mobile phases were used with the mAb Pac™ SCX-10 RS column (50 mm × 4.6 mm, 5 µm) and a generic gradient from 0 to 100% B in 30 min. To evaluate the performance with the two different buffer compositions, infliximab and three bispecific antibody products were analyzed, as these products represent the complexity of therapeutic proteins that are currently under development and contain strong charge heterogeneity with significant differences in size and p*I*s.

**Figure 1:**
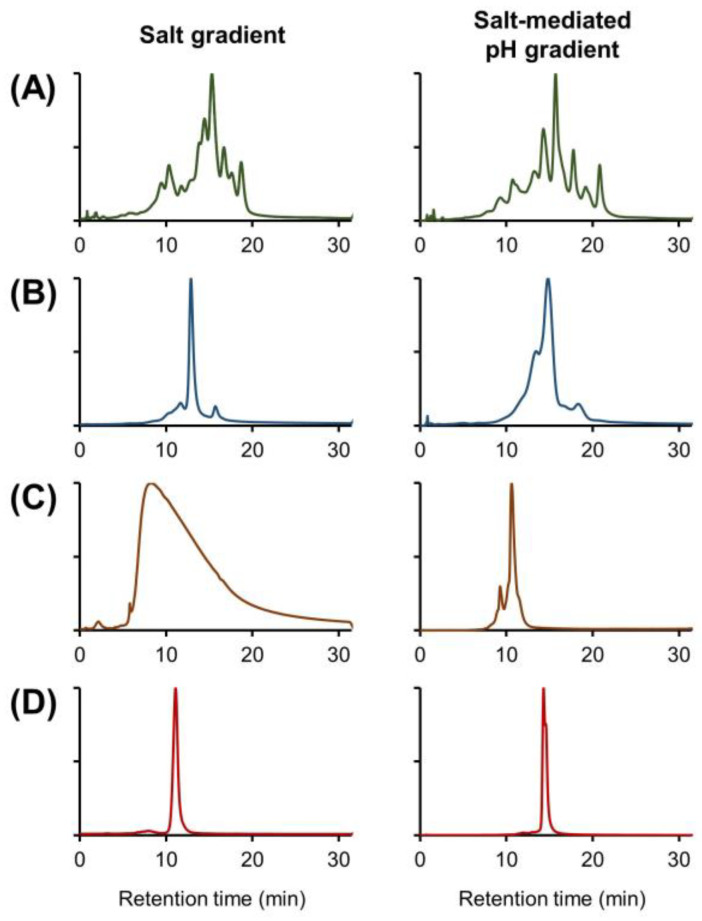
Comparison of salt gradient (**left panels**) and salt-mediated pH gradient (**right panels**) IEX analysis of infliximab and several complex mAb-based products. Chromatograms are displayed for infliximab (**A**), bsAb1 (**B**), bsAb2 (**C**), and bsAb3 (**D**). Intensities in EU normalized. Chromatographic conditions are described in Section 3.4.

After the initial scouting gradients, individual optimization of the gradient programs was performed for each product. The results for both buffer compositions are displayed in Figure 1 and serve as a reference and starting point for optimization in this study.

It was observed that with the salt gradient, the complex charge profile of infliximab (Figure 1A) was well resolved and that the salt-mediated pH gradient separated fewer charge variants, which is consistent with the recent literature [32]. For bsAb1 (Figure 1B), more charge species could be detected when using the salt-mediated pH gradient, and they can be explained by the fact that these gradient conditions are better adapted to products with high p*I* values [18]. For bsAb2, no charge species could be separated using the salt gradient, and a large, tailed peak was observed (Figure 1C). By using the alternative salt-mediated pH gradient, resolution was significantly increased, and an acidic and basic species could be partially separated from the main peak. For bsAb3, a near isocratic salt gradient (i.e., 28–30% B) was used and did not allow the separation of any charge variants (Figure 1D). When the salt-mediated pH gradient was used, only a basic species could be partially separated from the main peak. 

Overall, it was observed that the salt-mediated pH gradient was the best solution for the bsAbs species, while the salt gradient was suitable for the analysis of the canonical mAb product infliximab.

### 2.2. Evaluation of Solvent Addition in IEX Mobile Phase Buffer Compositions

To evaluate the influence of solvents in the mobile phase compositions for CEX, the analysis of infliximab using a salt-mediated pH gradient (0–100% B) with solvent-enriched mobile phases was performed. Due to the large amount of charge species present in the profile of infliximab, the effect of the solvent on retention and selectivity could be easily tracked. The following solvents were tested: IPA, butanol, dioxane, acetone, EtOH, ACN, and DMSO. The selected solvent concentrations varied from 20% *v*/*v* for IPA, EtOH, acetone, and ACN down to 5% *v*/*v* for butanol and dioxane and 1–5% *v*/*v* for DMSO. 

**Figure 2:**
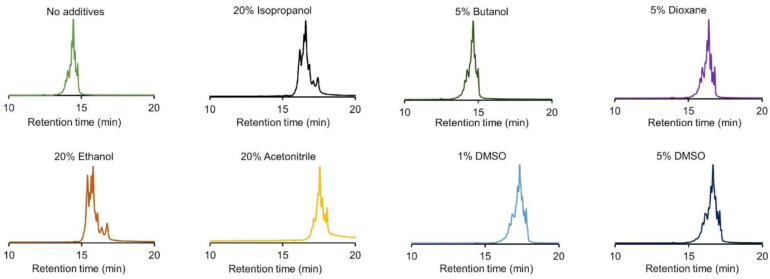
CEX chromatograms obtained for the analysis of infliximab using a solvent-enhanced salt-mediated pH gradient. Intensities in EU normalized. Chromatographic conditions are described in Section 3.4.

These concentrations were carefully selected to mitigate the potential for protein denaturation and salt precipitation in the high-ionic strength mobile phase B. Figure 2 shows the corresponding results for the analysis of infliximab with the enriched mobile phase compared to the normal salt-mediated pH gradient without solvent addition. It was observed that when acetone was added to the mobile phase, no peaks could be detected and therefore, acetone was excluded from further studies, most probably due to a lack of solubility of the protein in this solvent. With the reference conditions, elution of peaks occurred between 13 to 15 min, and various acidic and basic charge species were separated from the main peak. However, when solvents such as IPA, dioxane, EtOH, ACN, and DMSO were added to the mobile phase, the retention of all peaks shifted to later retention times. This shift suggests that the protein had a higher affinity for the stationary phase, resulting in increased retention. This effect was most prevalent when ACN was added to the mobile phase (average increase in retention time by more than 20%). When butanol was added, no apparent shift in retention occurred, and peaks eluted similarly to the unenriched mobile phase composition (peaks elution between 13 and 15 min). More importantly, the mobile phases enriched with IPA, EtOH, and ACN exhibited improved selectivity, as highlighted by a greater separation observed between the main peak and the various basic species. This was not achieved with the other solvents (i.e., butanol, dioxane, and DMSO), where peaks appeared in a similar elution window as with the not enriched mobile phase composition.

### 2.3. Use of IPA As Mobile Phase Additive in CEX

**Figure 3:**
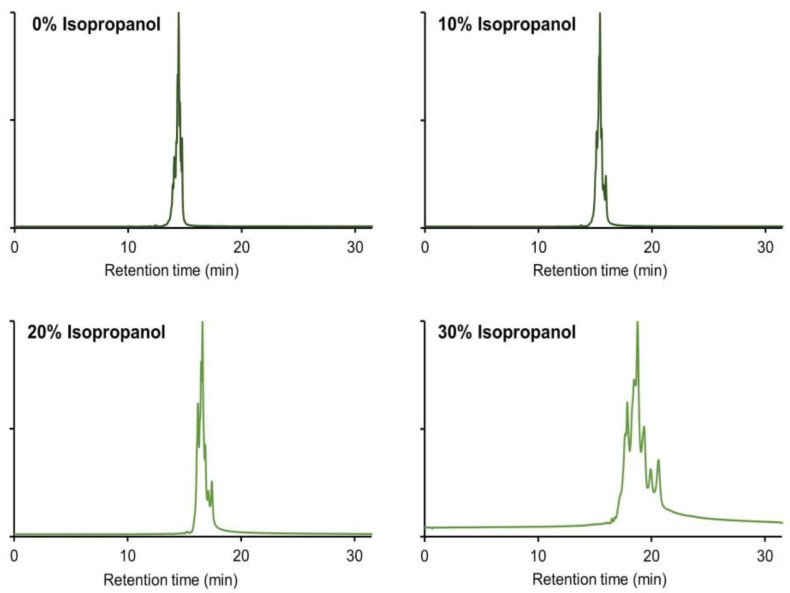
Influence of the increasing IPA content on the separation of infliximab charge variants. Intensities in EU normalized. Chromatographic conditions are described in Section 3.4.

From the investigated solvents allowing modification of selectivity and retention, IPA was selected and systematically studied in further detail. It was previously shown that the inclusion of a polar protic solvent like IPA in the mobile phase composition could effectively mitigate the secondary hydrophobic interactions with the stationary phase in size exclusion chromatography [43]. IPA is also known as one of the least denaturing solvents that can be used in chromatography. Additionally, it was reported that smaller quantities of IPA were needed compared to a polar aprotic solvent like ACN [43]. Hence, IPA was used in subsequent experiments to minimize the addition of large solvent amounts. This approach aimed to prevent the formation of artifacts and minimize the risk of protein denaturation associated with high solvent concentrations. Concentrations of IPA up to 40% in the mobile phase were tested and evaluated by analyzing infliximab with a generic 0–100% B gradient. It was observed that the main peak eluted at about 14.5 min under normal conditions and at 18.8 min when 30% *v*/*v* of IPA was added to the mobile phase (Figure 3). Higher concentrations than 30% *v*/*v* IPA were undesirable as no peaks were detected when 40% *v*/*v* of IPA was used, most probably due to the limited solubility of infliximab at this composition. Another benefit of the addition of IPA was the improvement in selectivity between the separated charge variants. Indeed, a significant improvement in selectivity was observed between the main peak and the basic variants upon the addition of the organic modifier. For example, the distance between the main isoform and the most basic variant ranged from 0.3 min for the standard conditions to 1.9 min for the mobile phase with 30% IPA, showing a higher number of separated peaks. 

**Figure 4:**
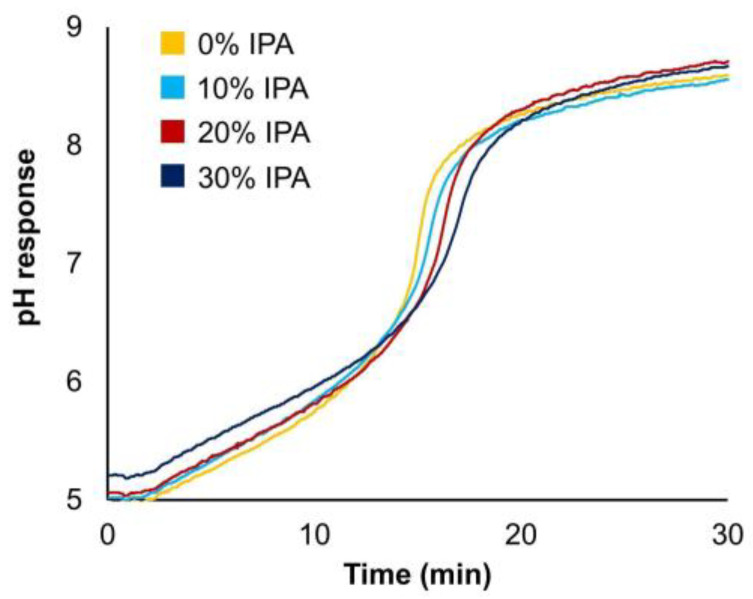
Influence of IPA content on the pH response during IEX separation. Chromatographic conditions are described in Section 3.4.

To assign this effect specifically to IPA, the pH response during the separation was monitored online and displayed in Figure 4. It was demonstrated that the effect of IPA on the pH response was minimal, and no discernible difference occurred between the separations conducted with and without an organic modifier. So, a modification of pH during analysis due to the presence of IPA is not the main reason for the improved selectivity, although care should be taken when measuring the pH in the presence of additives as the accuracy of the measurement could be compromised. The introduction of IPA to the mobile phase leads to an increase in on-column pressure due to the higher viscosity of this solvent. Indeed, during the CEX separation, the pressure drop was, in general, equal to 80 bar when IPA was not used, while it increased up to 150 bar when only 20% *v*/*v* of IPA was added to the mobile phase. Since this increase in on-column pressure could potentially affect the separation performance, as already reported in the literature, [44] several tests were performed using a column outlet restrictor.

**Figure 5:**
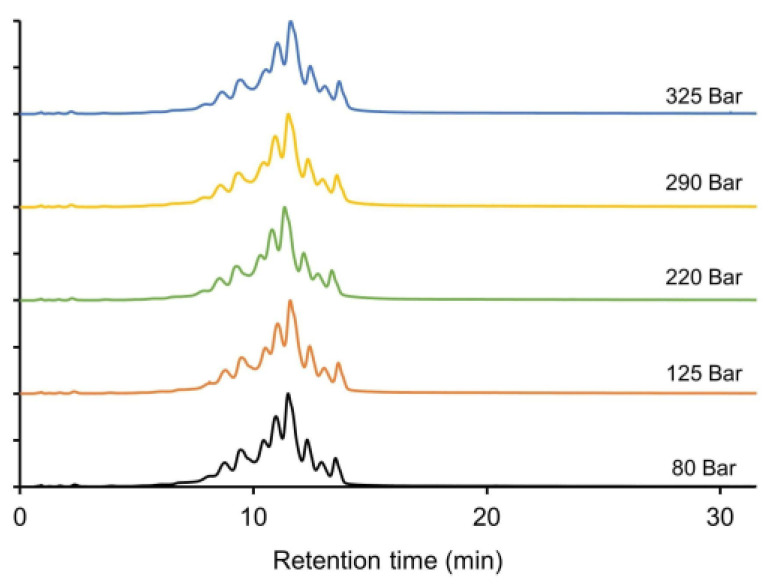
Influence of pressure on the separation of infliximab charge variants. Intensities in EU normalized. Chromatographic conditions are described in Section 3.4.

This allowed us to mimic the pressure rise that occurs when IPA is added to the mobile phase while excluding the use of the organic solvent itself. Figure 5 shows the results obtained after analyzing infliximab at different pressure conditions (80, 125, 220, 290, and 325 bar). The separation of the charge variants was closely examined under increased pressure conditions, and no discernible differences were observed. This finding suggests that the enhanced selectivity achieved by the addition of IPA cannot be attributed to the increase in pressure, and it can be concluded that no protein denaturation occurs because of the pressure changes. 

Another possible reason for the changes in selectivity observed in the presence of an organic solvent in CEX is a potential change in protein solvation. The latter is directly related to the hydration shell, which refers to the layer of water molecules that surround the analyzed molecule in an aqueous solution. When ionic molecules (such as proteins) are dissolved in water, they attract water molecules through electrostatic interactions and hydrogen bonds, forming a hydration shell around them.

The hydration shell plays a crucial role in ion exchange separations because it affects the accessibility to the charges of the proteins and, therefore, its chromatographic behavior. When an organic solvent is added to the mobile phase, it can modify the hydration shell and alter its dynamics, structure, and stability. The observed changes depend on several factors, including the nature of the solvent, the protein being analyzed, and their respective interactions with water molecules. In most cases, the organic solvent may compete with water molecules for the solvation of the proteins. Depending on the relative affinities of the proteins for water and the organic solvent, the organic solvent molecules can replace the water molecules in the solvation shell. This can lead to changes in the composition of the hydration shell. 

IPA has a lower polarity and dielectric constant compared to pure water. When small, mildly hydrophobic monohydric alcohols, such as IPA, are used, an alcohol-rich layer can be formed around the protein [45]. To assess whether the changes in selectivity observed in the presence of IPA can be attributed to changes in solvation, we attempted to analyze other model proteins having different physico-chemical properties, either more hydrophilic or more hydrophobic than infliximab. For this purpose, our recent work on hydrophobic interaction chromatography (HIC), which classifies commercial therapeutic mAbs according to their hydrophobicity, was considered [46]. 

**Figure 6:**
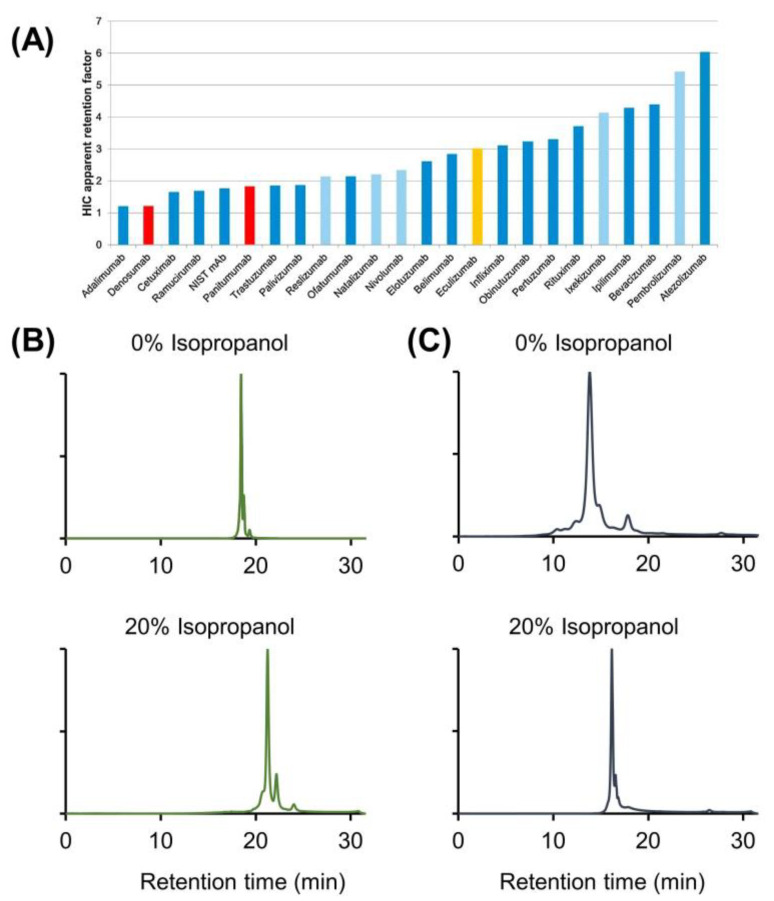
Effect of IPA on the separation of charge variants of mAbs with different hydrophobicities. HIC apparent retention factors adapted from Goyon et al. [46], Copyright 2017, with permission from Elsevier, (**A**) and effects of IPA on adalimumab analysis (**B**) and pembrolizumab analysis (**C**). Intensities in EU normalized. Chromatographic conditions are described in Section 3.4.

Based on Figure 6A, reporting the HIC apparent retention factors of different mAbs, adalimumab was selected as a representative hydrophilic mAb while pembrolizumab was selected as a hydrophobic mAb. We then performed a CEX analysis without the addition of IPA to the mobile phase and another analysis in the presence of 20% *v*/*v* IPA. In all cases, the gradient was generic (0–100% B). The corresponding chromatograms are shown in Figure 6B,C for adalimumab and pembrolizumab, respectively. In the case of adalimumab, the selectivity was modified in a positive way in the presence of IPA, with better discrimination of the two basic charge variants eluting after the main isoform, as well as the appearance of a peak shouldering in the region where the acidic variants are eluting. On the other hand, the opposite situation was observed for pembrolizumab, with more significant changes in the selectivity but an overall reduction in the separation quality. In this case, the basic variant, which was very well separated from the main isoform without IPA, was poorly resolved in the presence of IPA. In addition, a very strong overall decrease in selectivity was observed in the region where the acidic variants eluted. These results, obtained under strictly identical CEX conditions but with protein samples having substantially different hydrophobicity, demonstrate that the addition of IPA to the mobile phase in CEX can have a different impact (either positive or negative) depending on the hydrophobicity of the biopharmaceutical product.

The observed changes in chromatographic behavior are most probably due to a change in the solvation of mAbs in the presence or absence of IPA, with more or less hydrophobic proteins having different hydration in relation to their different physico-chemical properties. In the case of adalimumab, which contains numerous hydrophilic functional groups accessible to the mobile phase used in CEX, hydration occurs through interactions between these functional groups and water molecules. Conversely, pembrolizumab, which is more hydrophobic and, therefore, contains fewer hydrophilic functional groups, tends to be less hydrated in the presence of pure water. When adding a certain amount of IPA in the mobile phase, the alcohol can disrupt the interactions between the water molecules and the less polar regions of the proteins, which can lead to partial desolvation of these regions, resulting in possible conformational changes of the proteins, potentially altering CEX retention and selectivity. This behavior was observed for the reference mAbs considered in Figure 6B,C. 

However, it is important to note that protein hydration remains a complex phenomenon that depends on many factors, including the amino acid sequence, three-dimensional structure, and the physico-chemical environment of the proteins, making the results hardly interpretable.

### 2.4. Addition of Additives to Solvent Enriched Mobile Phases

To further investigate the impact of the mobile phase composition on selectivity, several mobile phase additives were stochastically tested both alone and in combination with the use of IPA and/or DMSO. Additives considered were NH_4_F, TFE, and HFIP. Generic gradients (0–100% B) were first performed using a salt-mediated pH gradient in the presence of the desired organic modifier. As reported in Figure S1, corresponding to the analysis of infliximab, similar results (in terms of selectivity) were obtained independently of the additive used in combination with 30% *v*/*v* IPA. In these conditions, the charge profiles consisted of around seven peaks (according to the visible apexes), although a return to baseline was compromised by signal drift. Such a signal drift after the elution of peaks can be attributed to the possible adsorption phenomenon. However, when using mobile phases enriched with 1% *v*/*v* DMSO and 50 mM HFIP, this issue was not identified, and up to nine apexes were visible (although less clearly resolved). In addition, a strong shift in retention was observed (main peak eluted 10 min earlier), denoting the possibility of eluting the infliximab charge variants by using a lower salt ionic strength. Despite this being of interest, the same behavior was not found during the analysis of the more complex bsAbs samples (Figures S2–S4), meaning that the mobile phase composition in CEX is not generic and should be optimized case by case. 

For the analysis of bsAb1 (Figure S2), the separation was not successful when using mobile phase additives in combination with 30% *v*/*v* IPA, as strong on-column protein adsorption was observed. Interestingly, the problem does not seem to be related to the use of IPA but rather to the combination of IPA and additives. In fact, testing mobile phases containing 1% *v*/*v* DMSO in combination with 50 mM HFIP (without IPA) allowed the proper elution of the protein, similar to the use of mobile phases containing 20% *v*/*v* IPA alone. Better peak shapes were observed for the mobile phases containing 1% *v*/*v* DMSO in combination with 50 mM HFIP and were, therefore, selected for further optimization. 

As for the analysis of bsAb2 (Figure S3), a better resolution was obtained when using mobile phases doped with 30% *v*/*v* IPA and 50 mM HFIP, while the use of 1% *v*/*v* DMSO and 50 mM HFIP let to the loss of selectivity and severe peak broadening and tailing. On the contrary, when analyzing bsAb3 (Figure S4), the same selectivity was obtained independently of the additive used in combination with 30% *v*/*v* IPA. Despite this, the use of 1% *v*/*v* DMSO in combination with 50 mM HFIP highlighted the presence of a charged variant as a post-peak shoulder that was partially resolved when using 1% *v*/*v* DMSO alone as a mobile phase additive. 

**Figure 7:**
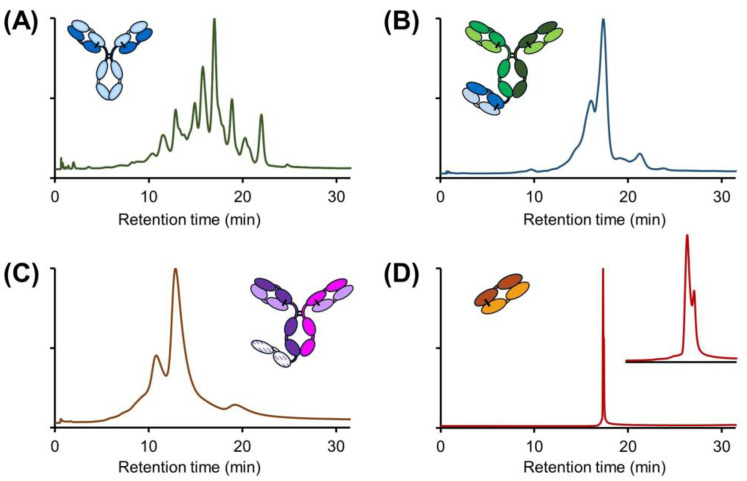
Optimized chromatograms obtained for infliximab (**A**), bsAb1 (**B**), bsAb2 (**C**)**,** and bsAb3 (**D**) using solvent and additive enriched salt-mediated pH gradients. Intensities in EU normalized. Gradient conditions described in Section 3.4.

Finally, the best mobile phase compositions were used to perform the CEX analyses with optimized linear gradients. As reported in Figure 7, these conditions corresponded to 40–50% B for the analysis of infliximab, 33–48% B for the analysis of bsAb1, and 55–80% B for the analysis of bsAb2. The only exception was the analysis of bsAb3, for which the best peak resolution was obtained when using a generic linear gradient from 0 to 100% B (the resolution of the peaks was lost when trying to focus the gradient). Of note, the optimization of the gradient conditions (Figure 7) allowed us to better resolve the charge variants as compared to the results obtained when using the not enriched mobile phases in salt-mediated pH gradient mode.

For example, the charge profile of infliximab consists of 14 peaks (according to the visible apexes) versus about 10 previously obtained. Similarly, up to five peaks (versus three previously obtained) were detected for the IEX profile of bsAb1.

Although the interpretation of these results is complicated by the fact that there is no common tendency that could facilitate the use of generic gradient conditions, it should be noted that the objective was to evaluate the possibility of improving the selectivity of resolved (or resolvable) IEX species by changing the composition of the mobile phase. This stochastic study on additives has highlighted some interesting mobile phase compositions (never reported before in the literature) that deserve to be taken into consideration in the future during the development of analytical methods for the analysis of complex samples such as bsAbs.

### 2.5. Are Solvent- and Additive-Enriched Salt-Mediated pH Gradients Stability Indicating?

**Figure 8:**
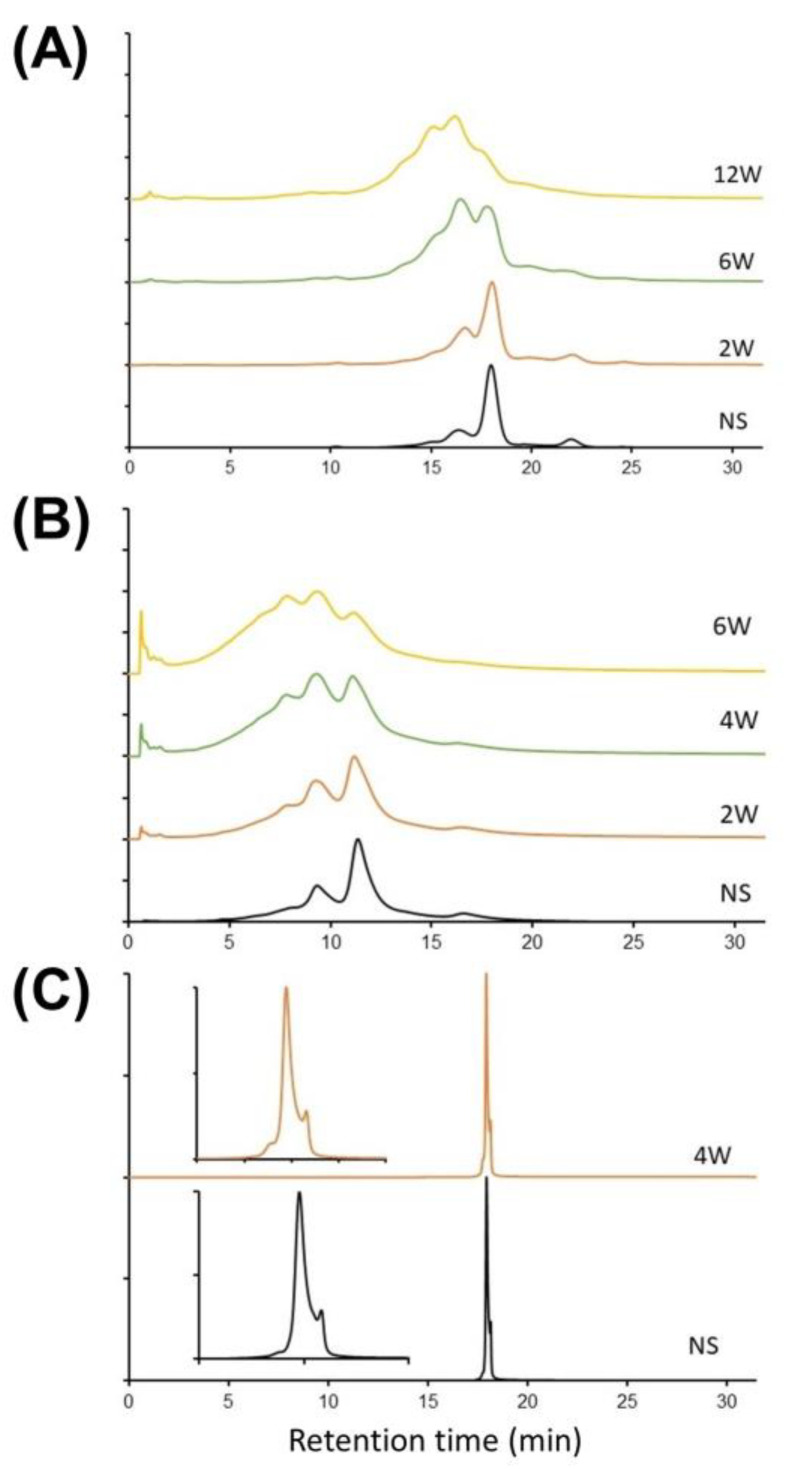
Stability test on thermally stressed bsAb1 (**A**), bsAb2 (**B**), and bsAb3 (**C**) by using solvent- and additive-enriched salt-mediated pH gradients. Intensities in EU normalized. Gradient conditions are described in Section 3.4.

The next step was to verify whether the IEX methods developed for the three bispecific antibodies could be considered as stability indicating. For this purpose, bsAb1, bsAb2, and bsAb3 samples were thermally stressed at 40 °C for 12, 6, and 4 weeks, respectively, and then analyzed by IEX solvent- and additive-enriched salt-mediated pH gradients. The results are shown in Figure 8. 

In the case of bsAb1 (Figure 8A), degradation remains minor after 2 weeks of thermal stress, but the charge profile deteriorates significantly after 6 weeks. In fact, the amount of acidic variants was much higher after 6 weeks of thermal stress. It was observed that the area of the peak eluting at about 16 min on the unstressed sample increases sharply after 6 weeks. In the region of the basic variants, the variant clearly separated on the unstressed sample (elution at 22 min) is eluted in the tail of the main peak after 12 weeks of thermal stress and is no longer distinguishable. In summary, there is a very significant broadening of the peaks after 12 weeks due to the presence of multiple charge variants that are poorly separated from the main isoform. It is, therefore, clear that the developed method is able to detect this instability. 

Figure 8B shows the results obtained for the bsAb2 sample. Again, the same type of behavior is observed, with increasingly pronounced differences in the IEX profiles as the exposure time of the sample to thermal stress increases. Indeed, the amount of acidic variants increases significantly with time, while the basic variant eluted at 16 min for the unstressed sample is lost in the tail of the peak observed after 6 weeks of exposure. For this sample, it is important to note that we did not observe this basic variant in Figure 1C when the mobile phase contained no organic solvents or other additives. This contrasts with the analysis of the bsAb1 sample shown in Figure 1B and Figure 8A, where a basic variant was already observed. Therefore, the question arises whether this additional basic variant observed on the bsAb2 sample could be due either to an increase in selectivity in the presence of IPA and/or HFIP or to degradation/denaturation of the sample under these analytical conditions. To answer this question, we attempted to identify this basic variant using high-resolution mass spectrometry (HRMS). Unfortunately, it appears that MS sensitivity was greatly reduced in the presence of HFIP and IPA due to sample denaturation (higher charge state) and the insufficient quality of the solvents/additives used. Under these conditions, it was not possible to identify this variant, and further work would be required to demonstrate the value of using these new additives/solvents in IEX-MS, which is beyond the scope of the present work. On the other hand, it is plausible that the denaturation induced by the analytical conditions could be at the origin of the appearance of this new basic variant since a change in the conformation of the bispecific antibody could lead to a different interaction with the IEX stationary phase and the apparition of a new peak with a higher exposed charge state (eluted in the basic variant region). 

Finally, Figure 8C illustrates the results obtained when analyzing bsAb3. Only one stressed condition was available for this sample (4 weeks). The chromatographic profile remains relatively consistent between the unstressed conditions and conditions where the sample was subjected to heat stress at 40 °C for 4 weeks. The amount of the basic variant remains strictly similar, while an acidic variant appears partially separated from the main isoform (this acidic variant appears as a shoulder of the main peak). Although the selectivity was limited for the separation of bsAb3, we can see an evolution of the sample over time, demonstrating that the method can be considered as stability indicating.

## 3. Materials and Methods

### 3.1. Chemicals and Reagents

Ultrapure water was obtained from a MilliQ purification system from Millipore (Bedford, MA, USA). Dimethyl sulfoxide (DMSO), acetonitrile (ACN), ethanol (EtOH), butanol, and propan-2-ol (IPA) were LC-MS grade (Optima^®^) and obtained from Fisher Chemical (Reinach, Switzerland). Acetic acid glacial (≥99.0%), ammonium hydroxide (28.0% NH_3_ in H_2_O, ≥99.99%), 1,4-Dioxane (>99%), 2-(N-Morpholino) ethanesulfonic acid (MES) monohydrate (≥99.5%), sodium chloride (≥99.5%), ammonium fluoride (NH_4_F), 1,1,1,3,3,3-Hexafluoro-2-propanol (HFIP, LC-MS grade), and trifluoroethanol (TFE) were obtained from Sigma-Aldrich (Buchs, Switzerland). Sodium hydroxide 1 M (1 N) in aqueous solution was from VWR (Nyon, Switzerland).

Therapeutic IgG monoclonal antibodies, adalimumab, infliximab, and pembrolizumab were obtained as European Union pharmaceutical-grade drug products from their respective manufacturers. Additionally, more complex mAb-related samples, including a mAb-domain-fusion (~200 kDa, bsAb1), a mAb-cytokine-fusion (~165 kDa, bsAb2), and a Fab domain (~50 kDa, bsAb3) were provided by Roche (Penzberg, Germany). All intact mAbs and the complex samples were diluted in pure water at 1 mg/mL.

### 3.2. Instrumentation and Columns

Measurements were performed on an ACQUITY UPLC H-Class Bio system (Waters, Milford, MA, USA) equipped with a quaternary solvent delivery pump, an autosampler including a 15 µL flow-through-needle injector, and a fluorescence (FL) detector (excitation at 280 nm, emission at 350 nm, 2 Hz) including a 2 µL low dispersion analytical flow-cell. In addition, an online pH meter (Monitor pH/C-900) equipped with a pH electrode (General Electric, Baden, Switzerland) was added at the column outlet to monitor pH for each separation. Measurements with restricting tubing to enhance the pressure were performed on an ACQUITY UPLC system (Waters, Milford, MA, USA) equipped with a binary solvent delivery pump and an autosampler with a fixed loop injector (2 µL). Different pressures were obtained by adjusting the length of a PEEKsil (I.D. 50 µm) restrictive tubing installed after the column. Data acquisition and instrument control were performed by Empower Pro 3 software. The strong cation exchange column mAbPac™ SCX-10 RS (50 mm × 4.6 mm, 5 µm) dedicated to mAbs and associated variants was purchased from Thermo Fisher Scientific (Reinach, Switzerland). This column was chosen after a preliminary screening was performed to check the impact of different column hardware on the pH response (Figure S5).

### 3.3. Mobile Phase Preparation

For classical salt gradient IEX experiments, mobile phases were composed of 20 mM MES at pH 5.6 (mobile phase A) and 20 mM MES + 0.5 M NaCl at pH 5.6 (mobile phase B). Further experiments were performed using a salt-mediated pH gradient in which the buffer system was composed of 50 mM ammonium acetate at pH 5 (mobile phase A) and 160 mM ammonium acetate at pH 8.5 (mobile phase B). To maintain the ionic strength consistent when adding the organic modifiers, the buffers were prepared by mixing a solution of 50 mM ammonium hydroxide prepared with the desired organic modifier (e.g., 20% IPA or 1% DMSO) with a solution of 50 mM acetic acid that is prepared with the desired organic modifier (e.g., 20% IPA or 1% DMSO) to reach the pH of 5. A similar approach was taken for the solutions containing an ionic strength of 160 mM ammonium acetate at pH 8.5. For the additives, a similar strategy was applied, and the following concentrations were used: 50 mM for HFIP, 2 mM NH_4_F, and 20 mM TFE.

### 3.4. Chromatographic Conditions

Both salt gradients and salt-mediated pH gradients were performed by running a linear gradient from 0 to 100% B for 30 min, after which the mobile phase composition was set back to 0% B for 15 min to equilibrate the column before injecting the next sample. An injection volume of 5 µL was used unless stated otherwise, the flow rate was set at 400 µL/min, and a column temperature of 25 °C was used. After an initial scouting experiment, the gradients were adapted for each sample separately to achieve the best possible separation of the charge variants. For the experiments performed with the pressure-restricting tubing, an injection volume of 2 µL was used with the fixed loop injector setup. Optimized salt gradients were achieved in 30 min with 15–30% B for infliximab, 16–25% B for the bsAb1, 0–100% B for the bsAb2, and 28–30% B for bsAb3. For the salt-mediated pH gradients, the optimized gradients were also achieved in 30 min with 35–42% B for infliximab, 50–60% B for the bsAb1, 45–70% B for the bsAb2, and 50–56% B for bsAb3. For the comparison of the organic modifiers, a standard 0–100% B gradient was used unless stated otherwise. Finally, solvent-enriched mobile phase composition used for the salt-mediated pH gradients consisted of the enrichment of the buffer system, composed of 50 mM ammonium acetate at pH 5 (mobile phase A) and 160 mM ammonium acetate at pH 8.5 (mobile phase B), with 1% *v*/*v* DMSO and 50 mM HFIP for infliximab (40–50% B) and bsAb1 (33–45% B), 30% *v*/*v* IPA and 50 mM HFIP for bsAb2 (55–80% B), and 1% *v*/*v* DMSO for bsAb3 (0–100% B).

## 4. Conclusions

This work set out to evaluate the use of solvent-enriched mobile phase composition for the IEX analysis of charge variants present among a canonical mAb product and multiple complex bsAb products. First, a comparison was made between the classical salt gradient and the ammonium-based salt-mediated pH gradient. The results demonstrated that while the salt gradient was effective for the canonical mAb product, infliximab, it had limitations when applied to the more complex bsAb samples, as fewer charge species could be identified. Then, multiple solvents were explored as a means to enhance the selectivity of the salt-mediated pH gradient. From the tested solvents, it was found that when IPA, dioxane, EtOH, ACN, and DMSO were added to the mobile phase, the retention of all peaks increased. From these solvents, IPA, EtOH, and ACN also exhibited slightly improved selectivity between the observed charge variants. Further investigation into the utilization of solvents, IPA in particular, revealed that the observed effect was unrelated to changes in pH response or the increased pressure during analysis. Instead, it appeared to be associated with the solvation layer surrounding proteins during the analysis and was different among mAb products with a strong hydrophilic character or hydrophobic character. It was observed that these products react differently to the presence of IPA. Finally, CEX analysis in the presence of various mobile phase additives, including NH_4_F, TFE, and HFIP, was tested alone or in combination with IPA and/or DMSO. For infliximab, the results showed similar selectivity regardless of the additive used with 30% *v*/*v* IPA. However, when 1% *v*/*v* DMSO and 50 mM HFIP were added to the mobile phase, improved resolution and a shift in retention were observed. In the analysis of the bsAbs, the optimal mobile phase composition varied depending on the specific bsAb being analyzed, and ultimately, optimized mobile phase compositions were determined for each antibody product, resulting in improved resolution compared to unenriched mobile phases.

Although no generic conditions could be determined for the analysis of the bsAbs, the findings offer valuable insights and demonstrate that when analyzing complex biopharmaceutical proteins, the use of solvent-enriched mobile phases can be an important parameter to consider during method development to improve selectivity. However, the compatibility of such IEX methods with mass spectrometry remains to be evaluated.

## Data Availability

Data is contained within the article and Supplementary Material.

## Supplementary Materials

The following supporting information can be downloaded at: https://www.mdpi.com/article/10.3390/ijms242316623/s1.
