# Supplementary material for: Enhancing Selectivity of Protein Biopharmaceuticals in Ion Exchange Chromatography through Addition of Organic Modifiers
Source: Int J Mol Sci. 2023 Nov 22;24(23):16623. doi: 10.3390/ijms242316623 (PMC10706461; doi:10.3390/ijms242316623)

Supporting Information

Enhancing selectivity of protein biopharmaceuticals in ion exchange chromatography through addition of organic modifiers

Bastiaan L. Duivelshof [1,2,†], Thomas Bouvarel [1,2,†], Sebastian Pirner [3], Vincent Larraillet [3], Alexander Knaupp [3], Hans Koll [3], Valentina D'Atri [1,2,*] and Davy Guillarme [1,2]

[1] School of Pharmaceutical Sciences, University of Geneva, CMU - Rue Michel Servet 1, 1211 Geneva, Switzerland
[2] Institute of Pharmaceutical Sciences of Western Switzerland, University of Geneva, CMU - Rue Michel Servet 1, 1211 Geneva, Switzerland
[3] Roche Diagnostics GmbH, Nonnenwald 2, 82377 Penzberg, Germany
* Correspondence: valentina.datri@unige.ch
† These authors contributed equally to this work.

Table of contents

Figure S1 Chromatograms of Infliximab: (A) 30% *v/v* IPA with 2 mM NH_4F; (B) 30% *v/v* IPA with 20 mM TFE; (C) 30% *v/v* IPA with 50 mM HFIP; (D) 1% *v/v* DMSO with 50 mM HFIP…..…… S1

Figure S2 Chromatograms of bsAb1: (A) 30% *v/v* IPA with 2 mM NH_4F; (B) 30% *v/v* IPA with 20 mM TFE; (C) 30% *v/v* IPA with 50 mM HFIP; (D) 1% *v/v* DMSO with 50 mM HFIP; (E) 20% *v/v* IPA…..…… S1

Figure S3 Chromatograms of bsAb2: (A) 30% *v/v* IPA with 2 mM NH_4F; (B) 30% *v/v* IPA with 20 mM TFE; (C) 30% *v/v* IPA with 50 mM HFIP; (D) 1% *v/v* DMSO with 50 mM HFIP…….…… S2

Figure S4 Chromatograms of bsAb3: (A) 30% *v/v* IPA with 2 mM NH_4F; (B) 30% *v/v* IPA with 20 mM TFE; (C) 1% *v/v* DMSO alone; (D) 30% *v/v* IPA with 50 mM HFIP; (E) 1% *v/v* DMSO with 50 mM HFIP…… S2

Figure S5 Evaluation of pH response with the MabPac 5 µm 2.1 x 50 mm column (green), the SEPAX SCX-NP5 Proteomix 5 µm 4.6 x 100 mm column (orange), the BioPro IEX SF 5 µm 4.6 x 100 mm column (blue), the BioResolve SCX 3 µm 4.6 x 50 mm column (red), the ProPac Elite WCX 5 µm 4 x 150 mm column (grey), and without column (yellow). pH was measured on-line on the H-Class Bio system equipped with an on-line pH meter when running a generic gradient from 0 %B to 100 %B in 30 minutes....…… S3

Figure S1: Chromatograms of Infliximab: (A) 30% *v/v* IPA with 2 mM NH_4F; (B) 30% *v/v* IPA with 20 mM TFE; (C) 30% *v/v* IPA with 50 mM HFIP; (D) 1% *v/v* DMSO with 50 mM HFIP.

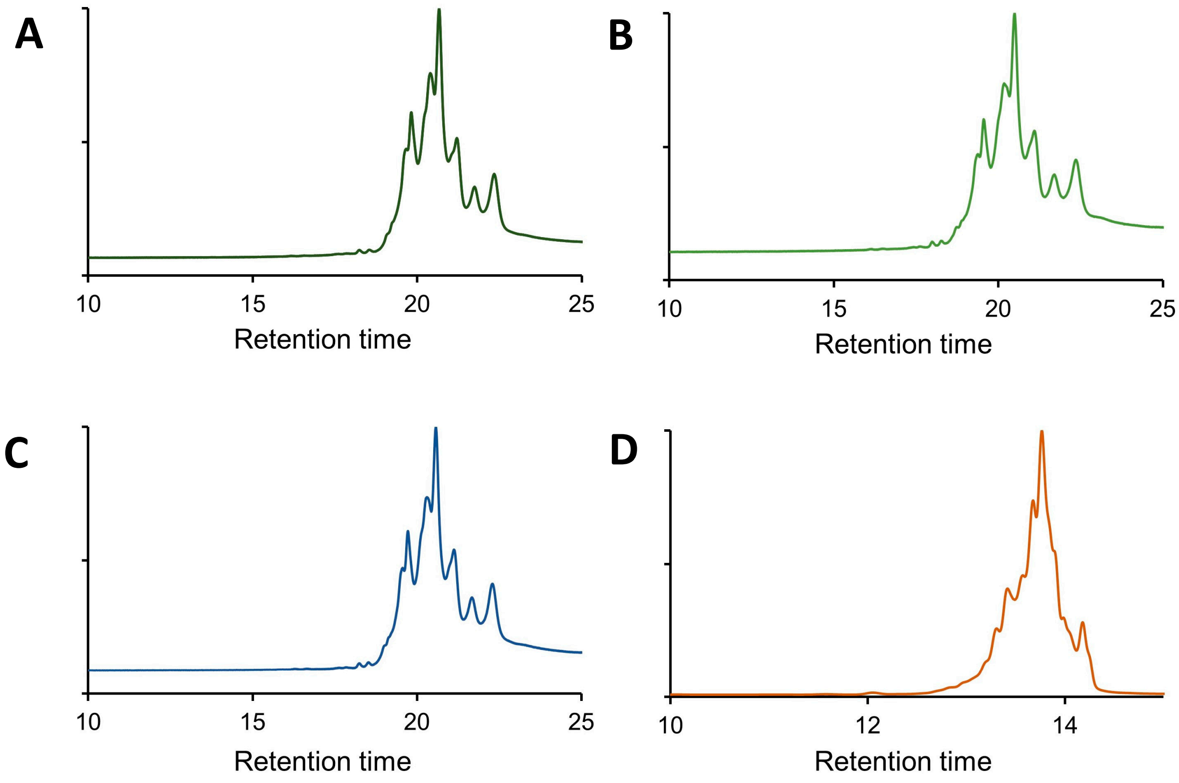

Figure S2: Chromatograms of bsAb1: (A) 30% *v/v* IPA with 2 mM NH_4F; (B) 30% *v/v* IPA with 20 mM TFE; (C) 30% *v/v* IPA with 50 mM HFIP; (D) 1% *v/v* DMSO with 50 mM HFIP; (E) 20% *v/v* IPA.

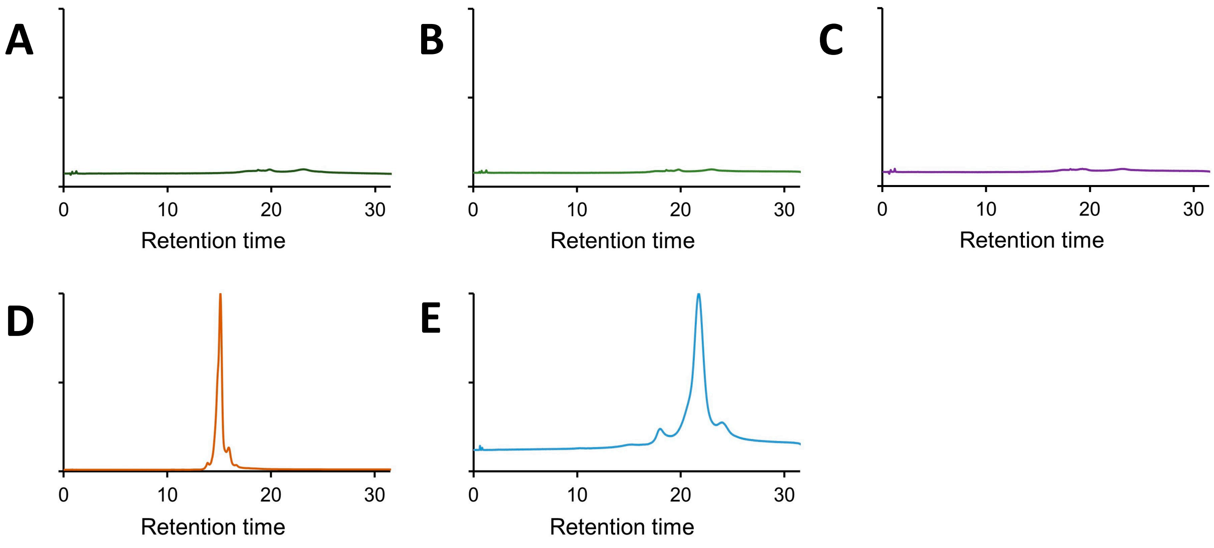

Figure S3: Chromatograms of bsAb2: (A) 30% *v/v* IPA with 2 mM NH_4F; (B) 30% *v/v* IPA with 20 mM TFE; (C) 30% *v/v* IPA with 50 mM HFIP; (D) 1% *v/v* DMSO with 50 mM HFIP.

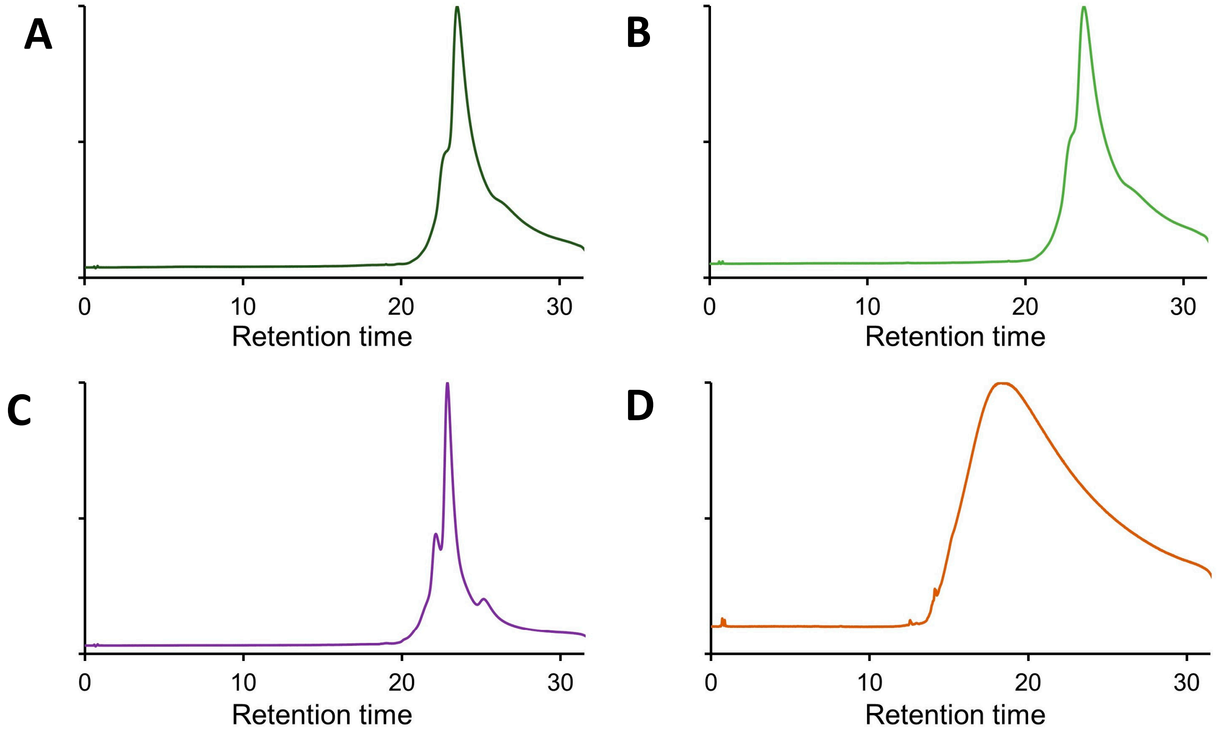

Figure **S4:** Chromatograms of bsAb3: (A) 30% *v/v* IPA with 2 mM NH_4F; (B) 30% *v/v* IPA with 20 mM TFE; (C) 1% *v/v* DMSO alone; (D) 30% *v/v* IPA with 50 mM HFIP; (E) 1% *v/v* DMSO with 50 mM HFIP.

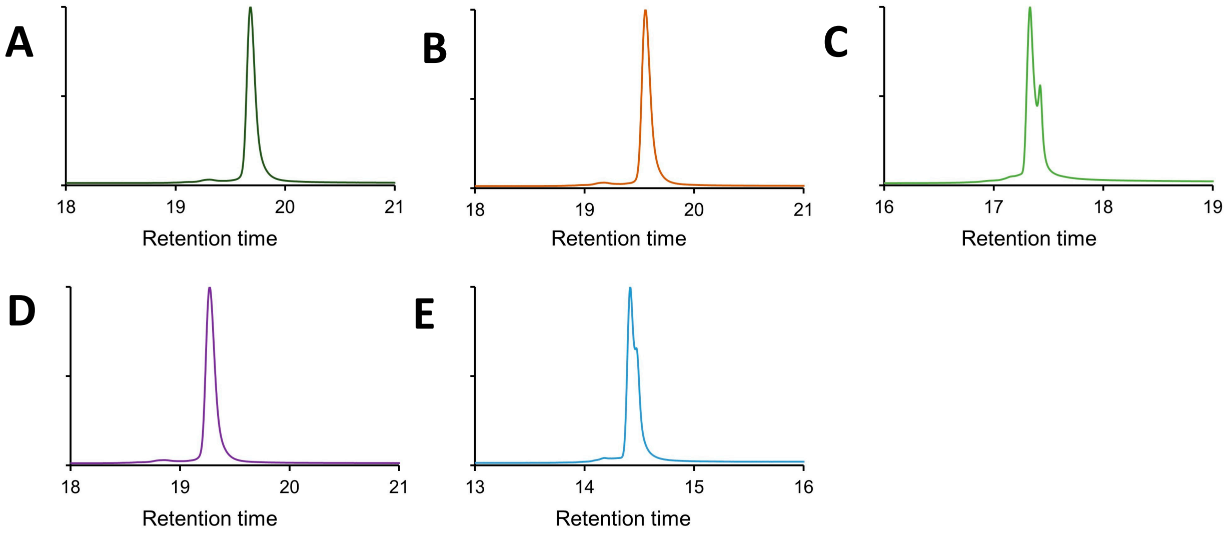

Figure S5: Evaluation of pH response with the MabPac 5 µm 2.1 x 50 mm column (green), the SEPAX SCX-NP5 Proteomix 5 µm 4.6 x 100 mm column (orange), the BioPro IEX SF 5 µm 4.6 x 100 mm column (blue), the BioResolve SCX 3 µm 4.6 x 50 mm column (red), the ProPac Elite WCX 5 µm 4 x 150 mm column (grey), and without column (yellow). pH was measured on-line on the H-Class Bio system equipped with an on-line pH meter when running a generic gradient from 0 to 100 %B in 30 minutes.

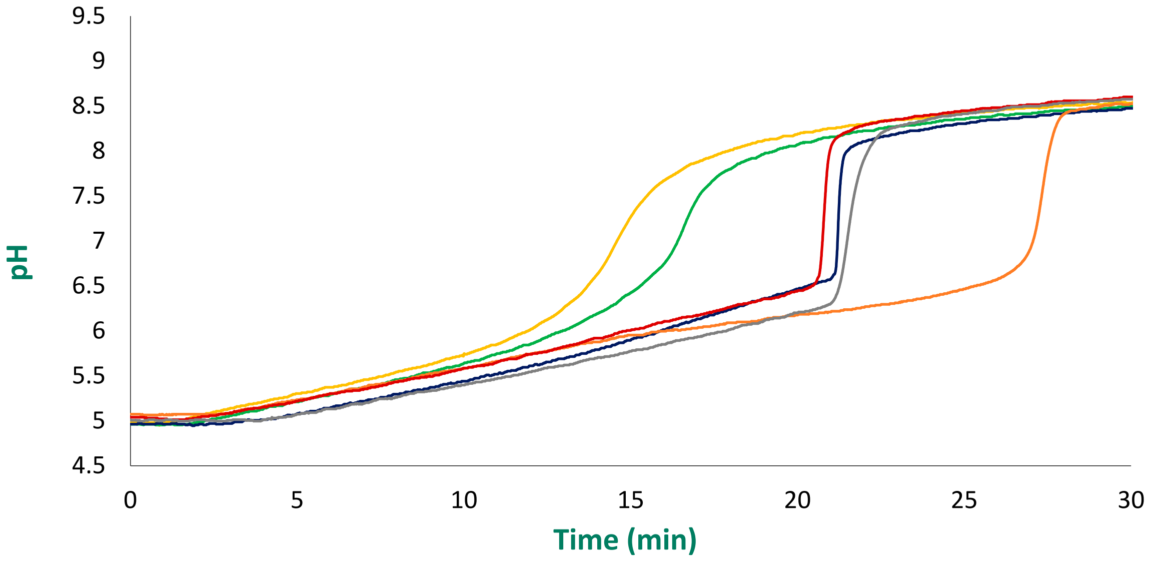

Supplement: Supplementary file 1 [file ijms-24-16623-s001.zip › ijms-2720513-supplementary.pdf]
